# Effects of β-Lactolin on Regional Cerebral Blood Flow within the Dorsolateral Prefrontal Cortex during Working Memory Task in Healthy Adults: A Randomized Controlled Trial

**DOI:** 10.3390/jcm10030480

**Published:** 2021-01-28

**Authors:** Yasuhisa Ano, Masahiro Kita, Keiko Kobayashi, Takashi Koikeda, Ryuta Kawashima

**Affiliations:** 1 Kirin Central Research Institute, Kirin Holdings Company, Ltd., 26-1, Muraoka-Higashi 2, Fujisawa Kanagawa 251-8555, Japan; Masahiro_Kita@kirin.co.jp (M.K.); Keiko_1_Kobayashi@kirin.co.jp (K.K.); 2 Shiba Palace Clinic, Daiwa A Hamamatsucho 6F, 1-9-10, Hamamatsucho, Minato-ku, Tokyo 105-0013, Japan; jimukyoku@mail.souken-r.com; 3 Institute of Development, Aging and Cancer (IDAC), Tohoku University, 4-1 Seiryo, Aoba-ku, Sendai 980-8575, Japan; ryuta.kawashima.a6@tohoku.ac.jp

**Keywords:** β-lactolin, cerebral blood flow, dorsolateral prefrontal cortex, near-infrared spectroscopy, whey, working memory

## Abstract

Epidemiological studies have reported that consumption of dairy products rich in β-lactolin is beneficial for cognitive decline among elderly individuals. Although previous studies have shown that β-lactolin supplementation improves memory function and attention in healthy adults, the mechanism through which β-lactolin affects human brain function has yet to be elucidated. This placebo-controlled randomized double-blind study therefore examined the effects of β-lactolin on human regional cerebral blood flow (rCBF) using near-infrared spectroscopy (NIRS) according to the Consolidated Standards of Reporting Trials guidelines. A total of 114 healthy participants aged between 50 and 75 years with relatively low cognition were randomly allocated into the β-lactolin or placebo groups (*n* = 57 for both groups) and received supplementation for 6 weeks. After the 6 weeks of supplementation, total hemoglobin during cognitive tasks (Kraepelin and 2-back tasks) was measured using two-channel NIRS to determine rCBF. Accordingly, the β-lactolin group had significantly higher changes in total hemoglobin at the left dorsolateral prefrontal cortex (DLPFC) area measured using the left-side channel during the 2-back tasks (*p* = 0.027) compared to the placebo group. The present study suggests that β-lactolin supplementation increases rCBF and DLPFC activity during working memory tasks.

## 1. Introduction

The number of individuals with cognitive decline or dementia has been rapidly increasing globally due to the increasingly aging society, with estimates predicting 130 million patients with dementia by 2050 [1]. Owing to lack of effective therapeutic approaches for cognitive decline and dementia, including Alzheimer’s disease, preventive approaches in daily life, such as exercise and food, have been gaining more attention. Accordingly, previous epidemiological investigations have reported that daily consumption of dairy products can prevent cognitive decline and decrease the risk for dementia [2,3,4,5], while others have demonstrated that dairy product intake prevents cognitive decline in rodents and humans [6,7]. Camembert cheese, a dairy product fermented using fungi, has been found to prevent the development of Alzheimer’s disease in transgenic model mice [8]. Reports have also revealed that Camembert cheese supplementation increased the brain-derived neurotrophic factor in the serum of patients with mild cognitive impairment [9].

Our group had previously identified β-lactolin, a β-lactoglobulin-derived glycine–threonine–tryptophan–tyrosine lacto-tetrapeptide of tryptophan-tyrosine-related β-lactopeptides abundantly found in fermented dairy products, including whey-enzymatic digestions, camembert cheese, and blue cheese [6,10]. Orally administered β-lactolin is delivered to the brain, where it inhibits monoamine oxidase B (MAO-B) activity in mice, subsequently increasing the levels of dopamine in the cortex and hippocampus [11,12]. β-Lactolin has been found to improve spatial working memory and episodic object recognition memory in mice and aged mice with pharmacologically induced amnesia [6], as well as improve cognitive decline and suppress Alzheimer’s pathologies, including inflammation and Aβ deposition in the brain of transgenic 5× FAD mice with Alzheimer’s disease [13].

Apart from preclinical studies, our previous randomized placebo-controlled trial showed that healthy middle-aged adults receiving β-lactolin supplementation for 6 weeks had better verbal fluency test scores evaluating executive function as well as better Stroop test scores evaluating inhibition of attention compared to the placebo group [14]. Based on this study, another randomized placebo-controlled trial also demonstrated that healthy older adults receiving β-lactolin supplementation had better sustained and selective attention evaluated using a visual-cancellation test and working memory evaluated using a paired-associate learning test compared to the placebo group [7]. It is also suggested that supplementation with β-lactolin increases the regional cerebral blood flow (CBF), but the number of participants was not enough to conclude the effects of β-lactolin on CBF [15]. The current study measured regional CBF after 6 weeks of β-lactolin supplementation. Previous publications have shown that β-lactolin supplementation improves memory, attention, and executive function, which have been especially associated with dorsolateral prefrontal cortex (DLPFC) function. Considering that impairment in DLPFC function has been associated with cognitive decline among elderly individuals and dementia, supplementation with β-lactolin can be expected to prevent cognitive decline and dementia. However, the association between β-lactolin supplementation with DLPFC activation has yet to be determined.

Cerebral nerve activity in the frontal cortex can be evaluated with a single-photon emission computed tomography (SPECT), magnetic resonance imaging (MRI), and near-infrared spectroscopy (NIRS). Given that NIRS is easy to use and non-invasive, the number of clinical trials using NIRS to measure DLPFC function in healthy participants has been increasing [16]. Unlike MRI and SPECT, NIRS can measure the CBF in the superficial cortex, the region of the DLPFC activated during β-lactolin supplementation. Recent studies using NIRS have been able to measure functional connectivity and cortical activation during a visuospatial working memory task, auditory working memory, and DLPFC activity during memory encoding and retrieval [17,18,19]. Studies have also shown that older adults receiving ginkgo extract supplementation had better cerebral perfusion as measured using SPECT compared to the placebo group [20], while others have found that ginkgo extract supplementation significantly increased changes in oxygenated hemoglobin (oxy-Hb) as measured using NIRS [21]. The aforementioned studies have shown that NIRS can be used for the evaluation of DLPFC activity. The present study conducted two randomized placebo-controlled trials wherein DLPFC activity during cognitive tasks was measured to investigate the effects of β-lactolin on DLPFC function. Total hemoglobin (Hb) during the cognitive tasks was measured using two-channel (2CH) NIRS after 6 weeks of intervention to explore DLPFC involvement during the cognitive tasks. The results obtained herein will certainly contribute toward elucidating the association between β-lactolin supplementation and DLPFC activation and CBF.

## 2. Materials and Methods

### 2.1. Participants

The present randomized, placebo-controlled, double-blind trial recruited 294 Japanese-speaking healthy older adults, aged 50 to 75 years, with subjective cognitive decline, such as forgetfulness and carelessness. Results other than NIRS measurements have been previously reported [7]. Participants with relatively low scores for the Repeatable Battery for the Assessment of Neuropsychological Status (RBANS) were preferentially included. Exclusion criteria included: (1) visual or hearing impediments, (2) suspected dementia, (3) anamnesis of cranial nerve disease, (4) diagnosis of depression or depressive symptoms, (5) menopausal symptoms or hormone treatment, (6) irregular lifestyle such as shift work, (7) high alcohol consumption (>20 g/day), (8) cigarette smoking, (9) experience of neuropsychological tests within 1 year, (10) current treatment for cognitive functions, (11) regular consumption of drugs or health foods affecting cognitive functions (>once a week), (12) regular consumption of protein supplements (>once a week), (13) anamnesis of severe disease requiring regular treatment, (14) allergy or sensitivity to milk, and (15) pregnancy or breastfeeding. Inclusion and exclusion criteria were assessed during screening using a questionnaire, Mini-Mental State Examination (MMSE), subject diary, clinical examination, and interview with the principal investigator.

### 2.2. Ethics and Registration

This trial was conducted in accordance with the Declaration of Helsinki and Ethical Guidelines for Medical and Health Research Involving Human Participants. Written informed consents were obtained from all participants. The study was approved by the Ethics Committee of Shiba Palace Clinic (Tokyo, Japan, chairman: Dr. Motoki Sano) (trial number: 138812-22675, title: A study for the effects of intake of whey enzymatic digestions on cognitive function in healthy older adults, a randomized controlled trial) and registered on 19 November 2017 in the database of the University Hospital Medical Information Network prior to enrollment (Registration No. UMIN000030461; Registration title. A study for the effect of intake of test foods on cognitive functions).

### 2.3. Experimental Supplements

Test tablets containing 1.6 mg of β-lactolin in whey enzymatic digestion were prepared by Kirin Holdings Co. Ltd. (Tokyo, Japan) as described previously [7]. The test tablets were ingested every day for 6 weeks. Meanwhile, placebo tablets were prepared by substituting whey peptides with the same amount of maltodextrin. Test and placebo tablets were indistinguishable in size, shape, and taste. The amount and duration of supplementation were similar to those utilized in previous trials considering that the current study aimed to investigate mechanisms involved in previous findings [7,14].

### 2.4. Procedures

This study employed a randomized, placebo-controlled, double-blind, parallel group comparative design. Questionnaire screening for inclusion/exclusion criteria and lifestyle; subject dairy evaluation; measurements of height, weight, and blood pressure; MMSE; and RBANS evaluation were performed upon initial screening. Thereafter, the principal investigator performed clinical examination for safety and eligibility in the second screening. Selected participants were randomly allocated to the placebo or β-lactolin group using a computer-based program to balance sex, age, and RBANS score. The person responsible for the group allocation has not been involved in the assessment of the eligibility, data collection, or analysis. Participants, research staffs, and outcome assessor were blinded to the allocation until analysis was completed. CBF measurements were performed after 6 weeks of intervention. Participants were instructed to keep their regular lifestyle and avoid any health functional food, drugs, or protein supplements that could affect cognitive function during the current study. Compliance was monitored with a diary. On the day of the neuropsychological tests, participants were instructed to completely avoid taking any food and beverages which contain caffeine and to avoid ingesting any food and beverages, except for water, 4 h prior to testing. Data were collected at Shiba Palace Clinic (Tokyo, Japan) between January 2018 and June 2018. This study was conducted by the contract research organization SOUKEN Co., Ltd.

### 2.5. Cerebral Blood Flow Measurements

**Figure 1:**
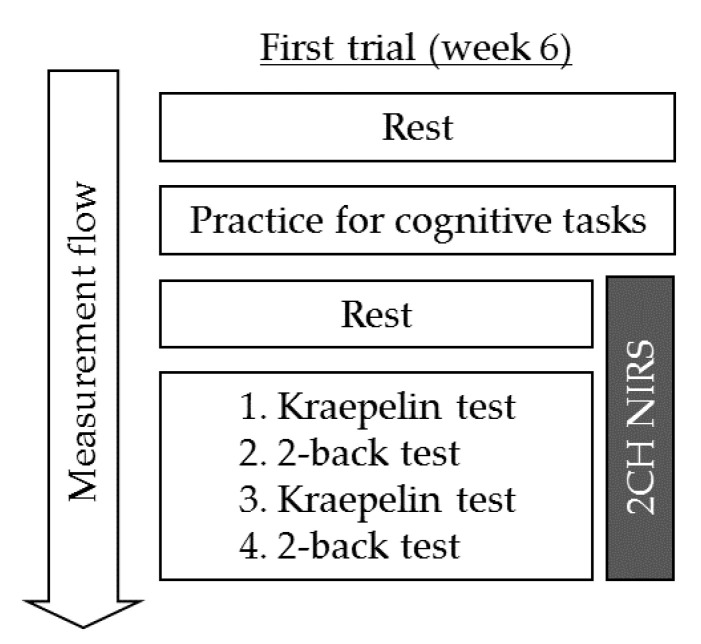
Measurement of regional cerebral blood flow. Regional cerebral blood flow (rCBF) during the Kraepelin and 2-back tasks was measured using two-channel near-infrared spectroscopy.

CBF was measured using 2CH NIRS after 6 weeks of intervention. Relative changes in the absorption of near-infrared light were measured at a time resolution of 10 Hz using a wearable 2CH NIRS system (HOT-1000, NeU Corporation, Tokyo, Japan), which employs a single light-emitting diode (810 nm) for the measurement of total Hb changes as demonstrated in a previous report [22]. NIRS probes were placed on either side of forehead in positions corresponding to the Fp1 and Fp2 areas including DLPFC area of the International 10–20 system. After fitting with the NIRS headset, instructions regarding the tasks were provided as subsequently described (Figure 1). Thereafter, participants completed a 5-min rest period, followed by task session completion. To determine hemodynamic response, concentration changes in total Hb were measured with a time resolution of 0.1 s. The moving average with 10-Hz sampling was applied. The pre-task baseline was determined as the mean value over 5 s of measurement immediately before the task. Mean pre-task baseline values were then subtracted from the mean task values. Thereafter, the average changes in total Hb concentration for each task were compared. Abnormal changes in total Hb concentration caused by motion artifacts were removed.

CBF was measured during the performance of the Kraepelin and 2-back tasks. In the Kraepelin task, which lasted for 3 min, participants were asked to calculate and write their answers within 30 s, after which they were directed to the next row of digits. The 2-back task required the participants to indicate whether the number presented on the screen was the same as the one presented two numbers back within a number sequence. Participants must respond by tapping the screen. This task lasted for 1.5 min, during which item hits, item correct rejections, and accuracy were scored. Tasks were presented in the following order: Kraepelin (3 min), 2-back (1.5 min), Kraepelin (3 min), and 2-back (1.5 min). Participants were given a 10-s resting period before each task periods. Kraepelin and 2-back are relatively easy cognitive tasks and used repeatedly in one day of the examination without leaning effects [23].

### 2.6. Statistical Analysis

Data are presented as means ± standard deviations (SD). We referred the previous plural statistical analysis using the fNIRS [24,25]. Comparisons between groups were conducted using unpaired *t*-tests as parametric test or Mann–Whitney U test as non-parametric test following the Kolmogolov–Smirnov test. All statistical analyses were performed using SPSS II for Windows (IBM Corp., Armonk, NY, USA), with *p* < 0.05 indicating statistical significance.

## 3. Results

### 3.1. Baseline Characteristics of the Study Group

**Figure 2:**
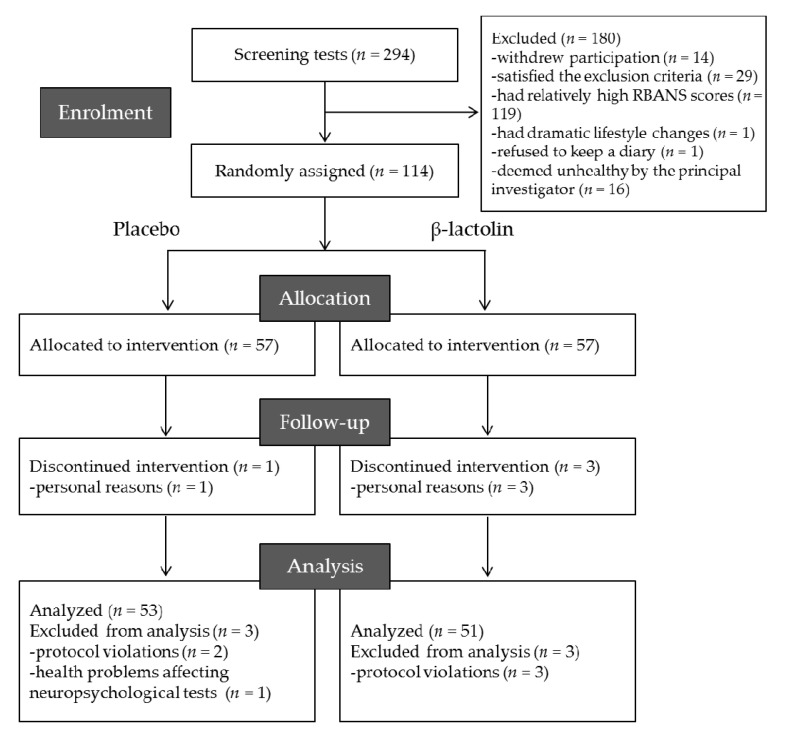
Consolidated standards of reporting trials diagram. Among the 294 participants screened, 114 were included. Participants were randomly allocated to placebo (*n* = 57) and β-lactolin (*n* = 57) groups. Four participants dropped out of the study, and 6 were excluded, leaving 53 and 51 participants in the placebo and β-lactolin groups for analysis, respectively.

A flowchart for participant inclusion is presented in Figure 2 in accordance with the previous report [7]. Following initial screening, 138 participants were included, whereas 156 participants were excluded because of withdrawal of participation (*n* = 6), suspected dementia (score of MMSE < 24; *n* = 5), regular consumptions of healthy foods affecting cognitive performance (*n* = 12), habit of high alcohol consumption (*n* = 3), dramatic change of lifestyle during screening (*n* = 1), irregular lifestyle (*n* = 8), experience of neuropsychological tests within 1 year (*n* = 1), refusal to keep a subject diary (*n* = 1), and relatively high score of RBANS (memory score > 50, *n* = 119). During the second screening, 24 participants were excluded due to classification as unhealthy by the principal investigator (*n* = 16) and withdrawal of participation (*n* = 8). The remaining 114 participants were randomly assigned into the β-lactolin or placebo group. One in the placebo group and three participants in the β-lactolin group withdrew from the study due to personal reasons. Three participants in the β-lactolin group were subsequently excluded from analysis due to violation of protocol (*n* = 3), while three participants in the placebo group were excluded due to protocol violation (*n* = 2) and health problems affecting neuropsychological tests (*n* = 1). Ultimately, 51 and 53 participants in the placebo and β-lactolin group, respectively, were analyzed, the characteristics of whom are summarized in Table 1.

### 3.2. Cerebral Blood Flow Measurement

**Figure 3:**
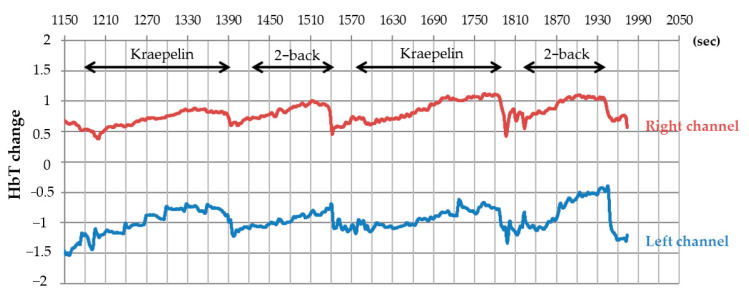
Representative total hemoglobin change during the working memory task. Total hemoglobin was monitored using a 2-channel NIRS. Representative change during the working memory tasks is shown in red (right channel) and blue (left channel)

After 6 weeks of intervention, CBF during the cognitive tasks was measured using 2CH NIRS (Figure 3). Accordingly, changes in total Hb concentration during the Kraepelin and 2-back tasks were measured, the results of which are presented in Table 2. The β-lactolin group had significantly higher changes in total Hb concentration within the left prefrontal cortex during the 2-back test (1st) than the placebo group (*p* = 0.027), although no such difference was observed for the right prefrontal cortex (Table 2). Moreover, the β-lactolin group had marginally significantly higher average changes in total Hb concentration for the 1st and 2nd 2-back test than the placebo group (*p* = 0.071). The number of item hits and accuracy did not differ between both groups (data not shown). Changes in total Hb during the Kraepelin task did not differ between both groups. 

## 4. Discussion

The current study investigated the effects of β-lactolin on CBF during a cognitive task with NIRS. Accordingly, our results showed that those receiving β-lactolin supplementation for 6 weeks had greater CBF within the DLPFC area during 2-back tasks compared to the placebo group, suggesting that β-lactolin supplementation promotes the activity of DLPFC during a cognitive task and is associated with the improvement of DLPFC-associated cognitive function, including attention, executive function, and memory retrieval.

The DLPFC is an area within the prefrontal cortex playing an essential role in executive function for cognitive process management [26], such as working cognitive flexibility [27], planning [28], memory, inhibition, and reasoning. Our previous randomized trials had demonstrated that β-lactolin supplementation improved executive function evaluated using a verbal fluency test, inhibition evaluated using the Stroop test, sustained and selective attention evaluated using a visual cancellation test [7], and associative working memory evaluated using the paired-associated test [7] in healthy older adults, all of which have been closely associated with DLPFC function. Studies have shown that damage to the DLPFC impairs working memory [29], attention [30], planning [31], and motivation to do things for oneself [32]. Moreover, DLPFC dysfunction has been associated with schizophrenia [33], depression [34], stress [35], and Alzheimer’s disease [36], as well as reduced CBF in the DLPFC, especially in the left DLPFC. Reports have found that elderly individuals exhibited impaired CBF in the left DLPFC and increased task errors during a card sorting test [37]. Furthermore, studies using SPECT have shown that CBF in the left DLPFC was associated with processing speed and perceptual reasoning in patients with moyamoya disease [38], while others have reported that *n*-back tasks were associated with DLPFC activation [39]. Pharmacological stress has also been shown to attenuate DLPFC activity and impair 2-back performance [40]. Recent meta-analysis showed that 2-back task increases activation in the left middle frontal gyrus, left inferior frontal gyrus, and left anterior insula [41]. Overall, previous studies have demonstrated that β-lactolin-induced increase in CBF within the left DLPFC area was associated with improved cognitive function among older adults [7,14,15]. The current study did not show the activation of CBF during the Kraepelin test. The Kraepelin test is a simple calculation task without memory function. Therefore, taking this into consideration along with previous trials, β-lactolin may show more effects on the cognitive tasks associated with memory function. Nonetheless, further studies using adequate cognitive task are needed to determine the mechanisms through which β-lactolin supplementation promotes neural activation, not only within the DLPFC but also in other frontoparietal areas.

Recent investigations have demonstrated that transcranial direct current stimulation of the DLPFC enhances inhibitory attention, working memory, and executive function among patients with Alzheimer’s disease [42,43,44]. Repeated stimulation to left DLPFC using transcranial magnetic stimulation also improved depressive and anxiety symptoms in medication-naïve patients with depression [45]. Although direct stimulations have not been approved as a therapy, they are required during surgery. Other safer approaches for the activation of DLPFC have gained increasing attention. A recent clinical trial reported that training of working memory improves CBF and functional connectivity during rest [46]. Moreover, clinical trials have identified certain nutrients that improve CBF in the frontal cortex. For instance, Ginkgo biloba supplementation among older adults has been found to promote CBF and cognitive performance [20]. Unfortunately, limited approaches have been available in promoting CBF within the DLPFC. As such, β-lactolin supplementation will be a new approach in promoting CBF within the DLPFC and improving cognitive function among elderly individuals.

The functions of the DLPFC are regulated by neurotransmitters, particularly dopamine distributing mostly in the frontal cortex area, which has been associated with its functions [47]. Considering that aging reduces dopamine levels in the frontal cortex area, which is associated with cognitive decline [48], treatments that increase dopamine levels in the cortex improve cognitive function. The levels of the dopamine D1 receptor, which is most involved in working memory tasks [49], in the parietal and frontal cortices have been found to reduce age-dependently, with such a reduction having been associated with impaired working memory ability [50]. Moreover, L-dopa ameliorates high-level cognitive deficits in patients with Parkinson’s disease by increasing CBF within the DLPFC, suggesting the involvement of the dopamine system in improving cognitive function [51]. Our previous preclinical investigations demonstrated that β-lactolin, orally administered, activates dopamine neurons and increases the levels of dopamine in the hippocampus and cortex, which improve object recognition and spatial memory object recognition memory in mice. β-lactolin inhibits MAO-B activities, increases dopamine levels in the cortex and the hippocampus, and improves cognitive decline [6,52]. Treatment with the dopamine D1 receptor antagonist attenuates the memory improvement by of β-lactolin in amnesia model mice. In addition, our previous preclinical studies had shown that the levels of dopamine in the cortex were reduced in a 5×FAD mice model of Alzheimer’s disease [13,53]. The aforementioned reports therefore suggest that β-lactolin improves the dopaminergic system and increases CBF within the DLPFC, promoting improvements in cognitive decline and dementia.

The current study has several limitations worth noting. CBF at week 0 of the intervention and during practices was not measured. Although numerous reports regarding rCBF did not include measurements at 0 weeks including randomized clinical trials [54,55], given that rCBF measurements are relative to those at baseline, there is a need to confirm baseline levels to definitively determine the effects of β-lactolin on rCBF. While the present study suggests that supplementation with β-lactolin enhances left DLPFC activity, the area was limited only to the left side. Moreover, a significant increase in CBF was observed only during the 2-back test, which requires maintaining, continuous updating, and processing of information. To definitively determine the effects of β-lactolin on CBF within the DLPFC during general working memory tasks, further studies including larger populations and employing not only NIRS but also MRI or SPECT are required. In addition, the current study evaluates participants with a lower score of RBANS, because the previous demonstration suggests that β-lactolin improves memory function with high cognitive fatigue [14]. To generalize our conclusions, further study is needed to evaluate the effects of β-lactolin on general older adults.

In conclusion, the present study indicated that β-lactolin supplementation increased rCBF in the left side of DLPFC during *n*-back test. Therefore, the previous clinical trials and the current study in tandem suggest that β-lactolin intake in daily life might improve rCBF and promote cognitive performance associated with the DLPFC.

## Figures and Tables

**Table 1:** Subject characteristics at baseline.

Characteristics	Placebo	β-Lactolin	*p*
Age	61.2 ± 5.6	60.7 ± 5.7	0.703
Male/female	20/33	17/34	0.639
BMI	22.1 ± 3.3	21.6 ± 2.8	0.414
MMSE	28.7 ± 1.4	28.5 ± 1.5	0.565
RBANS	54.5 ± 12.1	53.7 ± 14.1	0.772

Data are presented as means ± standard deviations (SD) for the placebo (*n* = 53) and β-lactolin groups (*n* = 51). The *p* value was calculated by unpaired *t*-tests, whereas those for male/female ratios were calculated using χ^2^ tests.

**Table 2:** Total hemoglobin measurement in the dorsolateral prefrontal cortex (DLPFC) using 2-channel near-infrared spectroscopy (CH NIRS). (**A**,**B**), changes in total hemoglobin (mM·mm) during the Kraepelin and 2-back tests in left and right channels measured using two-channel near-infrared spectroscopy, respectively.

(**A**)				
	**Group**	***n***	**Week 6**	***p***
Kraepelin (1st)	Placebo	47	0.29 ± 0.29	0.51
	β-lactolin	46	0.27 ± 0.32	
2-back (1st)	Placebo	45	0.073 ± 0.18	0.027
	β-lactolin	47	0.15 ± 0.16	
Kraepelin (2nd)	Placebo	47	0.23 ± 0.26	0.84
	β-lactolin	46	0.25 ± 0.31	
2-back (2nd)	Placebo	46	0.096 ± 0.17	0.42
	β-lactolin	47	0.14 ± 0.19	
Kraepelin (Average)	Placebo	47	0.26 ± 0.26	0.80
	β-lactolin	46	0.26 ± 0.30	
2-back (Average)	Placebo	46	0.086 ± 0.16	0.071
	β-lactolin	47	0.14 ± 0.15	
(**B**)				
	**Group**	***n***	**Week 6**	***p***
Kraepelin (1st)	Placebo	49	0.26 ± 0.23	0.11
	β-lactolin	44	0.18 ± 0.29	
2-back	Placebo	47	0.12 ± 0.19	0.73
	β-lactolin	46	0.13 ± 0.19	
Kraepelin (2nd)	Placebo	49	0.22 ± 0.30	0.24
	β-lactolin	44	0.15 ± 0.24	
2-back (2nd)	Placebo	48	0.15 ± 0.25	0.52
	β-lactolin	46	0.10 ± 0.15	
Kraepelin (Average)	Placebo	49	0.24 ± 0.25	0.14
	β-lactolin	44	0.16 ± 0.25	
2-back (Average)	Placebo	48	0.14 ± 0.20	0.62
	β-lactolin	46	0.12 ± 0.16	

Data are presented as means ± standard deviations (SD). The number of samples is represented in the table. Group differences were identified by unpaired *t*-tests.

## Data Availability

The data presented in this study are available on reasonable request from the corresponding author.
